# Unveiling the processing mechanism of Hezi-decoction-processed Tiebangchui: a synthesis approach using UPLC-Q-TOF-MS-based metabolomics and DESI-MSI

**DOI:** 10.3389/fphar.2025.1534748

**Published:** 2025-04-07

**Authors:** Zhen Zhou, Hui-Min Gao, Zhao-Qin Pei, Meng-Xiang Sha, Cong-Ying Li, Yi Zhang, Jin-Song Su, Yue Liu, Xian-Li Meng

**Affiliations:** ^1^ School of Ethmic Medicine, Chengdu University of Traditional Chinese Medicine, Chengdu, China; ^2^ School of Pharmacy, Chengdu University of Traditional Chinese Medicine, Chengdu, China; ^3^ Innovative Institute of Chinese Medicine and Pharmacy, Chengdu University of Traditional Chinese Medicine, Chengdu, China; ^4^ State Key Laboratory of Southwestern Chinese Medicine Resources, Innovative Institute of Chinese Medicine and Pharmacy, Chengdu University of Traditional Chinese Medicine, Chengdu, China

**Keywords:** Tiebangchui, UPLC-Q-ToF-MS, Hezi-decoction-processed, toxicity attenuation, DESI-MSI

## Abstract

**Introduction:**

Tiebangchui (TBC, Tibetan name: བང་ང་ནག་པ།), the dried tuberous root of *Aconitum pendulum* Busch. and *Aconitum flavaum* Hand.-Mazz., is a prevalent used Tibetan medicine, recognized for its significant therapeutic effects despite its high toxicity. It is commonly employed in treating the diseases categorized as “Long” (རླུང་ནད།), cold, “Huang-shui” (སེར་ཆུ་ནད།), leprosy, and mania in Tibetan medicine. Notably, it is utilized in the treatment of rheumatoid arthritis, which is classified under the “Huang-shui” disease category according to Tibetan medical theory. Given its considerable toxicity, various processing techniques aimed at reducing the harmful effects of TBC are essential for its safe application in clinical settings. Hezi-decoction-processed method is a distinctive and effective traditional processing method of Tibetan medicine, but the overall variability of chemical constituents in the Hezi-decoction-processed TBC is still unclear. This investigation sought to examine a variety of diterpenoid alkaloids and tanning constituents, identify potential metabolic markers for differentiating the unprocessed TBC and Hezi-decoction-processed TBC at varying processing times, and determine the optimal processing time for reducing toxicity and maintaining efficacy.

**Methods:**

A combination of metabolomic techniques was developed, integrating ultra-high-performance liquid chromatography-quadrupole time-of-flight mass spectrometry (UPLC-Q-TOF-MS) with desorption electrospray ionisation mass spectrometry imaging (DESI-MSI) coupled with quantitative analytical techniques. This was done with the objective of monitoring the dynamic alterations in chemical constituents in TBC during the processing time. Metabolic markers were observed via DESI-MSI, and three alkaloids and five tannin acids were quantified through the use of UPLC and HPLC.

**Results:**

Fifty-one compounds were identified in unprocessed TBC and processed samples, of which 31 were discernible from unprocessed TBC. A total of 22 metabolic markers, such as aconine, aconitine, benzoylaconine, chebulic acid, gallic acid, and corilagin, can proficiently distinguish between raw and processed TBC with different processing times. And the results of content determination of three alkaloids and five tannins showed that they were stabilized at 72 h. The monoester-diterpenoid alkaloids (MDAs) and diester-diterpenoid alkaloids (DDAs) levels were 0.0149% and 0.0852% in 72 h, respectively. The contents of gallic acid, corilagin, 1,2,3,4,6-*O*-pentagalloylglucose, chebulinic acid, and ellagic acid were 8.9706, 9.3444, 1.2438, 5.7582, and 3.1160 mg/g, respectively. The distribution and accumulation of metabolic markers during processing were investigated by DESI-MS. The results of DESI-MSI were consistent with those of content determination experiments. Combined with the multivariate statistical analysis, content determination of three alkaloids and five tannin acids and DESI-MSI, 72 h is demonstrated to be the appropriate time for toxicity attenuation and efficacy reservation of TBC.

**Discussion:**

The implementation of this technique could contribute to the identification of markers in Hezi decoction-processed TBC and the establishment of effective quality control and evaluation procedures to ensure the safety of TBC. The proposed method has the potential to elucidate the processing mechanism of *Aconitum* medicines and other toxic traditional Chinese medicines, given its wide applicability.

## Graphical Abstract

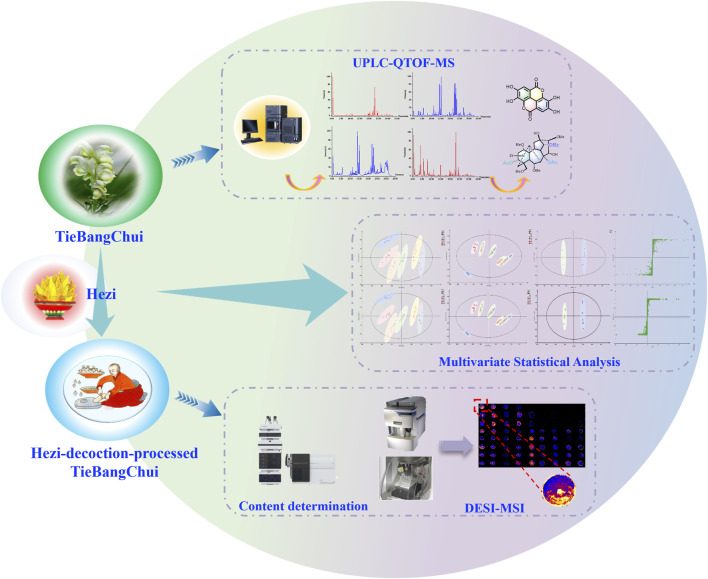

## 1 Introduction

The Tibetan medicine Tiebangchui (TBC, Tibetan name: བང་ང་ནག་པ།) is the dried tuberous root of *Aconitum pendulum* Busch. and *Aconitum flavaum* Hand.-Mazz. in the genus *Aconitum* of the Ranunculaceae family (Li et al., 2022; Li et al., 2024). The classic works of Tibetan medicine, Jing Zhu Materia Medica, document that TBC has a slight gas, bitter taste, and hemp. TBC therapeutic effects include the promotion of blood circulation and the dissolution of blood stasis, the dispersion of wind and the removal of dampness, and the attenuation of swelling and the alleviation of pain (Wang et al., 2016). TBC is widely used in treating “Long” (རླུང་ནད།), cold, “Huang-shui” (སེར་ཆུ་ནད།), leprosy, and mania in Tibetan medicine. Rheumatoid arthritis, which belongs to the “Huang-shui” disease category in Tibetan medicine theory, can be treated using the roots and seedlings of TBC. According to the *Four Volumes of Medical Treatment* (Yi Liao Si Bu Lun), “Huang-shui” is caused by the bile essence produced by the three tastes of the human body after the Stomach Fire digests, decomposes, and absorbs the nuances of food (Di-Si-Sang-Ji-Jia-Cuo, 1983; GYu-thogrNying-maYon-tanmGon-po, 1983). When the influence of multiple internal and external pathogenic factors leads to the imbalance of the three stomach fires in the human body, “Huang-shui” forms the Huang-shui disease. Pathological accumulation of “Huang-shui” in the joints can cause arthropathy, including rheumatoid arthritis (Su et al., 2023). TBC possesses significant toxicities, and improper usage may result in neurological, cardiovascular, and gastrointestinal irritation reactions, which usually manifest as heart pain, irregular heartbeat, numbness, and shortness of breath (Chan, 2016; Li S. et al., 2023). The primary constituents of TBC consist of *Aconitum* alkaloids, which encompass C_19_-, C_20_-, and various other bis-diterpenoid alkaloids (Li et al., 2022). These compounds were systematically categorized into three main categories based on their toxicity: high-toxicity diester-diterpenoid alkaloids (DDAs), moderate-toxicity monoester-diterpenoid alkaloids (MDAs), and nontoxic non-esterified diterpene alkaloids (NDAs). Among them, the specific compounds of DDAs are aconitine, 3-deoxyaconitine, mesaconitine and hypaconitine; MDAs such as benzoyl aconitine, benzoylmesaconitine and benzoylhypaconitine are well known; and the representative compounds of NDAs are aconine, mesaconine and hypaconine. These classes represent the active and toxic components of the alkaloids (Liu et al., 2017; Liu et al., 2022; Qiu et al., 2021).

TBC was characterized as “the head of the immovable poison” and underscores the necessity of processing to reduce its toxicity, according to “Jing Zhu Materia Medica” (Wang et al., 2010). Studies have shown that the alkaloid content responsible for toxicity changed significantly after processing, so processing methods that effectively attenuate toxicity and reserve the efficacy to use TBC are essential to ensure clinical safety (Wang Y. et al., 2010; Wang Y. J. et al., 2010). From ancient times to the present day, numerous records of processing methods of TBC can be found, among which the TBC processed with Hezi decoction is a special traditional one (Cao et al., 2024). Hezi (dried fruit of *Terminalia chebula* Retz.) is a widely used traditional Tibetan medicine, frequently designated the “king of medicines.” As a particularly good remedy for aconite poisoning, Hezi is widely employed in Tibetan medical formulations, including Shiwei Hezi San and Wuwei zuoxiang wan, due to its diverse medicinal activities and capacity for detoxification. The chebulic acid in Hezi could prevent the hydrolysis of aconitine during the brewing process and block TRPV1 channels, thereby resisting cardiotoxicity (Liu et al., 2023). Nevertheless, the overall variability of metabolites in Hezi-decoction-processed TBC is still unclear, and there is a notable absence of a unified investigation into the alterations in the chemical constituents of the botanical drugs corresponding to varying decoction durations. Therefore, revealing the main differential chemical metabolites in the concoction process and discovering their relationships with different concoction times could offer a scientific foundation for the control and optimization of the processed technique of TBC.

The complexity and variability of plant secondary metabolites is a major challenge in traditional Chinese medicine research, and traditional analytical methods can hardly meet the requirements of simultaneous analysis of different types of chemical components. Metabolomics is based on high-resolution and high-throughput detection platforms that comprehensively analyze metabolites in organisms and screen out metabolites with remarkable differences to study metabolic processes and change mechanisms (Tian et al., 2022). The fundamental principle of Chinese medicinal formulations lies in the alteration of chemical composition, and the use of metabolomics to search for potential metabolic differences between botanical drugs before and after preparations provides a material basis for the quality differences between Chinese medicine before and after preparations, which is conducive to finding the optimal preparations and elucidating the safety and scientificity of the preparations. Metabolomics has been widely used to study the changes in botanical drugs during manufacturing (Yang et al., 2023). For example, ultrahigh-performance liquid chromatography-quadrupole time-of-flight mass spectrometry (UPLC-Q-TOF-MS)-based metabolomics was employed for rapidly evaluating the distinctive metabolic profiles of red ginseng as well as ginseng prior to and following processing (Zhang et al., 2012). UPLC-Q-TOF-MS can also be linked to exogenous metabolomics for qualitative analysis of proximate constituents and metabolites, which can make the process of data analysis more accurate, simplify the process of compound resolution, and function as a valuable instrument for the screening and identification of metabolic constituents. Mass spectrometry imaging, which is a molecular imaging technique that is based on mass spectrometry, can be used to visualize thousands of compounds simultaneously without the need for labeling (Wu, 2023). Desorption electrospray ionization mass spectrometry imaging (DESI-MSI) integrates the analytical capabilities that mass spectrometry was combined with computer imaging for direct *in-situ* visualization of frozen tissue sections (Kumar, 2023). The composition, abundance, and *in-situ* spatial distribution of molecules on the surface of plant tissue sections can be obtained directly, providing important spatial information for plant metabolomics research. The integration of the two methodologies yields a thorough comprehension of the distribution and molecular mechanisms underlying compound biosynthesis, offering an effective method for visualizing the accumulation patterns of natural compounds as well as providing more significant and distinctive views of secondary metabolism.

The present study employed multivariate chemical analysis techniques of metabolomics linked to multivariate statistical analysis for exploring diterpenoid alkaloids and tanning constituents and to identify potential metabolic markers for distinguishing between TBC and Hezi-decoction-processed TBC at different processing times. In addition, the chosen metabolic markers were imaged and examined by DESI-MSI, and the levels of three indicator alkaloids and five tannin acids were determined by UPLC and HPLC. The results showed a significant difference between TBC made at different processing times and Hezi-decoction-processed TBC, and a processing time of 72 h was found to be conducive to the attenuation of toxicity and the preservation of efficacy. DESI-MSI coupled with metabolomics is an effective approach for observing altered patterns of diterpenoid alkaloids and identifying biomarkers in Hezi-decoction-processed TBC and detoxification treatment. The implementation of this technique could contribute to the identification of markers in Hezi decoction-processed TBC and the establishment of effective quality control and evaluation procedures to ensure the safety of TBC. Additionally, this approach enhances the understanding of the processing mechanisms associated with Aconitum and other toxic traditional Chinese medicinas.

## 2 Methods and materials

### 2.1 Chemicals and reagents

Formic acid and ammonium acetate of HPLC grade were procured from Chengdu Kelong Chemical Co., Ltd., (Chengdu, China). Acetonitrile and methyl alcohol of HPLC grade were supplied by Fisher Chemicals (Pittsburg, United States). Distilled water of HPLC grade was supplied by Watsons Distilled Water Co., Ltd., (Beijing, China). All other reagents were of analytical grade.

The reference standards (HPLC greater than 98%) of mesaconitine, benzoylaconine, benzoylhypacoitine, benzoylmesaconine, 3-acetylaconitine, fuziline, neoline, aconitine, 3-deoxyaconitine, hypaconitine, 12-epi-napelline, aconine, chebulic acid, gallic acid, rutin, chebulinic acid, ellagic acid and protocatechuic acid were purchased from Chengdu Push Biological Technology Co., Ltd., (Chengdu, China). Quercetin and corilagin (HPLC greater than 98%) were procured from Chengdu Must Biotechnology Co., Ltd., (Chengdu, China). 1,2,3,4,6-*O*-pentagalloylglucose (HPLC greater than 98%) was acquired from Chengdu Chroma Biotechnology Co., Ltd., (Chengdu, China).

### 2.2 Plant materials

Eight batches of TBC were collected from Huzhu Tu Autonomous County, Haidong City, Qinghai Province. Hezi was obtained from the Lotus Pond Market for Chinese Herbal Medicine in Chengdu. They were authenticated as dried root tubers of *A. pendulum* Busch. and dried ripe fruits of *T. chebula* Retz., respectively, by Prof. Yi Zhang (Chengdu University of Traditional Chinese Medicine).

### 2.3 Preparation of Hezi-decoction-processed TBC

Hezi (50.0 g) was soaked in deionized water (200 mL, at a weight-to-volume ratio of 1:4) for a duration of 70 min, followed by a boiling process lasting 30 min. Subsequently, Hezi decoction was cooled.

Raw TBC samples were randomly divided into eight groups (50.0 g for each group). One group was selected as raw TBC, whereas the remaining groups were soaked in Hezi decoction for 6, 12, 24, 48, 72, 96, and 120 h. Hezi decoction was replaced every day, and TBC was stirred during soaking.

### 2.4 UPLC-Q-TOF-MS analysis

#### 2.4.1 Preparation of sample solution

Samples of Hezi-decoction-processed TBC were dried at 45°C. Subsequently, the dried samples were filtered through a 65-mesh sieve after being powered. TBC sample powder (0.25 g) was measured and then subjected to extraction via ultrasonication using 25 mL of methanol for a duration of 30 min at a power of 300 W and a frequency of 40 kHz. Methanol was added to account for any weight loss incurred during the extraction process. Following extraction, the mixture underwent centrifugation for 5 min at 13,000 rpm, and the resulting supernatant was filtered using a membrane filter with a pore size of 0.22 μm.

#### 2.4.2 Preparation of standard solution

Reference standards comprising mesaconitine, benzoylaconine, benzoylhypacoitine, benzoylmesaconine, 3-acetylaconitine, fuziline, neoline, aconitine, 3-deoxyaconitine, hypaconitine, 12-epi-napelline, aconine, chebulic acid, gallic acid, rutin, chebulinic acid, ellagic acid, protocatechuic acid, quercetin, corilagin and 1,2,3,4,6-*O*-pentagalloylglucose were dissolved in methanol as a stock solution (1 mg/mL for each compound). The solution was mixed and diluted with methanol at a concentration of 10 μg/mL to prepare a mixed reference solution. The mixed standard solution was kept at 4°C.

#### 2.4.3 Chromatography and MS conditions

Data for UPLC-Q-TOF-MS were generated utilizing the Waters ACQUITY UPLC and SYNAPT XS HDMS systems (Waters Corporation, Milford, United States), which are outfitted with an electrospray ionization source and a hybrid Q-TOF mass spectrometer operating under the MSE model. The system was managed using Masslynx software (version 4.2). The chromatographic separation was performed using an ACQUITY UPLC BEH C_18_ column (2.1 mm × 100 mm, 1.7 μm). The mobile phase consisted of 0.1% formic acid in water (solvent A) and acetonitrile (solvent B), with a flow rate maintained at 0.3 mL/min. The gradient elution program was implemented as follows: 2%–7% B (0–1 min); 7%–11% B (1–3 min); 11%–15% B (3–8 min); 15%–30% B (8–13 min); 30%–36% B (13–16 min); 36%–70% B (16–20 min); 70%–85% B (20–26 min); 85%–85% B (26–28 min). The column temperature was set to 35°C. The injection volume was set at 2 μL.

The full-scan data were obtained in both positive and negative ion modes from 100 Da to 1,200 Da, with a scan duration of 0.3 s. The experimental parameters included a capillary voltage of 2000 V, an extraction cone voltage of 4 V, a source temperature of 150°C, a desolvation temperature of 450°C, a sample cone voltage of 40 V, a desolvation gas flow rate of 800 L/h, and a cone gas flow rate of 50 L/h. The lockspray scan duration was established at 0.3 s with a 30-s interval, and the data were averaged across three scans.

#### 2.4.4 Data processing

Data acquisition was conducted utilizing MassLynx software (version 4.2, Waters Corporation, Milford, United States). UNIFI (Waters Corporation, Milford, United States) and Progenesis QI (Waters Corporation, Milford, United States) were employed for the purposes of chromatographic peak extraction, alignment, and data normalization. Subsequently, the processed data were input into SIMCA-P 13.0 (Umetrics, Umea, Sweden) to facilitate multivariate data analysis.

### 2.5 DESI-MSI analysis

#### 2.5.1 Preparation of slices

All samples were directly sectioned, yielding cross-sectional slices with an approximate thickness of 5 mm. Following this, the samples underwent freeze drying at −80°C for a duration of 2 h. They were then affixed to glass slides and preserved at −80°C in preparation for DESI-MSI analysis.

#### 2.5.2 MS conditions

DESI-MS data were obtained utilizing a Waters Synapt XS HDMS Q-TOF mass spectrometer, which was outfitted with a DESI source (Waters Corporation, Milford, United States). The parameters were as follows: ionization mode was set to positive with a capillary voltage of 3.0 kV, a nebulizing gas pressure of 0.60 MPa, and a mass range spanning from 100 to 1,500. The spray solvent comprised 90% methyl alcohol supplemented with 0.2% formic acid and 0.1 mM leucine enkephalin delivered at 2 μL/min. For the negative ionization mode, the capillary voltage was adjusted to 2.0 kV, the nebulizing gas pressure was 0.65 MPa, and the mass range remained consistent at 100–1,500, utilizing the same spray solvent and flow rate as in the positive mode.

DESI imaging was conducted by high-definition imaging (HDI) software (version 1.5, Waters Corporation, Milford, United States). The mass spectrometry imaging was executed by processing the raw mass spectrometry files through the HDI software, employing LE as the lock mass for calibration of high-resolution mass spectrometry in both positive ionization mode (m/z 556.2772) and negative ionization mode (m/z 554.2620).

### 2.6 Determination of three alkaloid compositions in Hezi-decoction-processed TBC

#### 2.6.1 Preparation of sample solution

TBC sample powder (2.0 g) underwent ultrasonic extraction with 3 mL of ammonia and 50 mL of a 1:1 (v/v) mixture of isopropanol and ethyl acetate for 30 min at a power of 300 W and a frequency of 40 kHz. Following the extraction process, isopropanol-ethyl acetate (1:1, v/v) was added to compensate for any weight loss experienced during extraction, after which the mixture was filtered. Next, 25 mL of the resulting filtrate was accurately measured and evaporated to dryness under decreased pressure at 40°C. The residue was dissolved in 3 mL of 0.05 M hydrochloric acid–methanol solution. The supernatants obtained were utilized as the sample solution after undergoing centrifugation at 15,000 rpm for 5 min.

#### 2.6.2 Preparation of standard solutions

A mixed standard stock solution comprising benzoylaconine, aconitine, and 3-deoxyaconitine was diluted with 0.05 M of hydrochloric acid-methanol solution to generate six suitable concentrations for the development of calibration curves. The calibration curves were created by graphing the peak areas of the reference standards in relation to their corresponding concentrations. The mixed reference solution was kept at 4°C. For detection purposes, 2 μL of the mixed reference solution were injected into the UPLC system.

#### 2.6.3 UPLC conditions

The separation process was conducted utilizing the Waters ACQUITY UPLC system (Waters Corporation, Milford, United States), which included a binary pump, a mobile phase degasser, a temperature-controlled autosampler, a column thermostat, and a photodiode array detector. The chromatographic analysis utilized an ACQUITY UPLC BEH C18 column with dimensions of 2.1 mm × 100 mm with a particle size of 1.7 μm. The mobile phase comprised acetonitrile (solvent A) and water containing 0.2% glacial acetic acid (solvent B), with the pH adjusted to 6.5 using triethylamine. The gradient elution profile was established as 21%–29% B (0–3 min) and 29%–35% B (3–7 min). The flow rate was maintained at 0.4 mL/min, and the injection volume was set at 2 μL. The column temperature was regulated at 35°C, and UV detection was conducted at a wavelength of 235 nm.

### 2.7 Determination of main tannin acids in Hezi-decoction-processed TBC

#### 2.7.1 Preparation of sample solution

TBC sample powder (2.0 g) underwent ultrasonic extraction using 25 mL of methanol for a duration of 30 min, operating at a power of 300 W and a frequency of 40 kHz. Subsequent to the extraction process, additional methanol was incorporated to account for any weight loss incurred during extraction. The resultant extract solution was further filtered via a 0.22 µm membrane.

#### 2.7.2 Preparation of standard solutions

A mixed standard stock solution comprising gallic acid, corilagin, 1,2,3,4,6-*O*-Pentagalloylglucose, chebulinic acid, and ellagic acid was prepared and diluted with methanol to achieve six suitable concentrations for the development of calibration curves. Calibration curves were generated by plotting the peak areas of the reference standards against their respective concentrations. The mixed reference solution was kept at 4°C for storage. For detection purposes, the injection volume of the mixed reference solution was set at 10 μL.

#### 2.7.3 HPLC conditions

HPLC analyses were conducted utilizing a Shimadzu 2010 analytical HPLC system (Shimadzu Corporation, Kyoto, Japan), which included an LC-2010 pump, an LC-2010 column oven, an LC-2010 detector, and an LC-2010 autosampler. The separation procedure was performed using an Agilent ZORBAX Eclipse XDB-C_18_ column (4.6 × 250 mm, 5 µm). The mobile phase comprised 0.04% phosphoric acid solution (solvent A) and methanol (solvent B). The gradient elution profile was established as follows: 3%–7% B (0–12 min); 7%–20% B (12–20 min); 20%–30% B (20–50 min); 30%–35% B (50–70 min); and 35%–36% B (70–75 min). The flow rate was 1 mL/min, and the injection volume was set at 10 μL. The column temperature was maintained at 35°C. UV detection was conducted at a wavelength of 245 nm.

## 3 Results

### 3.1 Identification of metabolites accumulated in raw and Hezi-decoction-processed TBC

**FIGURE 1:**
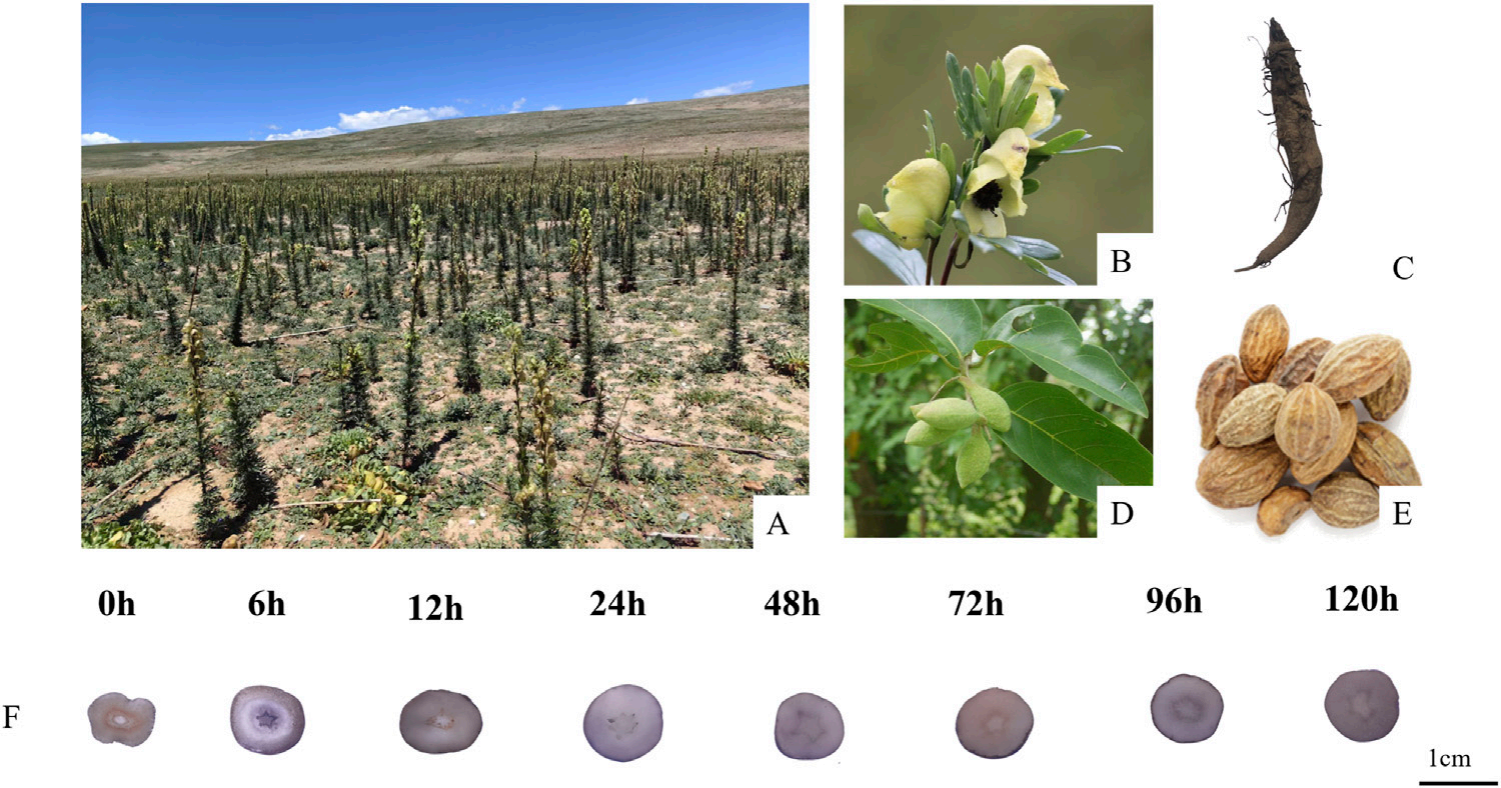
The original plants of *Aconitum pendulum* in the field **(A)**; the original plants of *Aconitum pendulum* **(B)**; the fresh roots of *Aconitum pendulum* **(C)**; the original plants of *Terminalia chebula* Retz. **(D)**; the dried ripe fruit of *Terminalia chebula* Retz. **(E)**; TBC processed with Hezi-decoction for different time points **(F)**.

**FIGURE 2:**
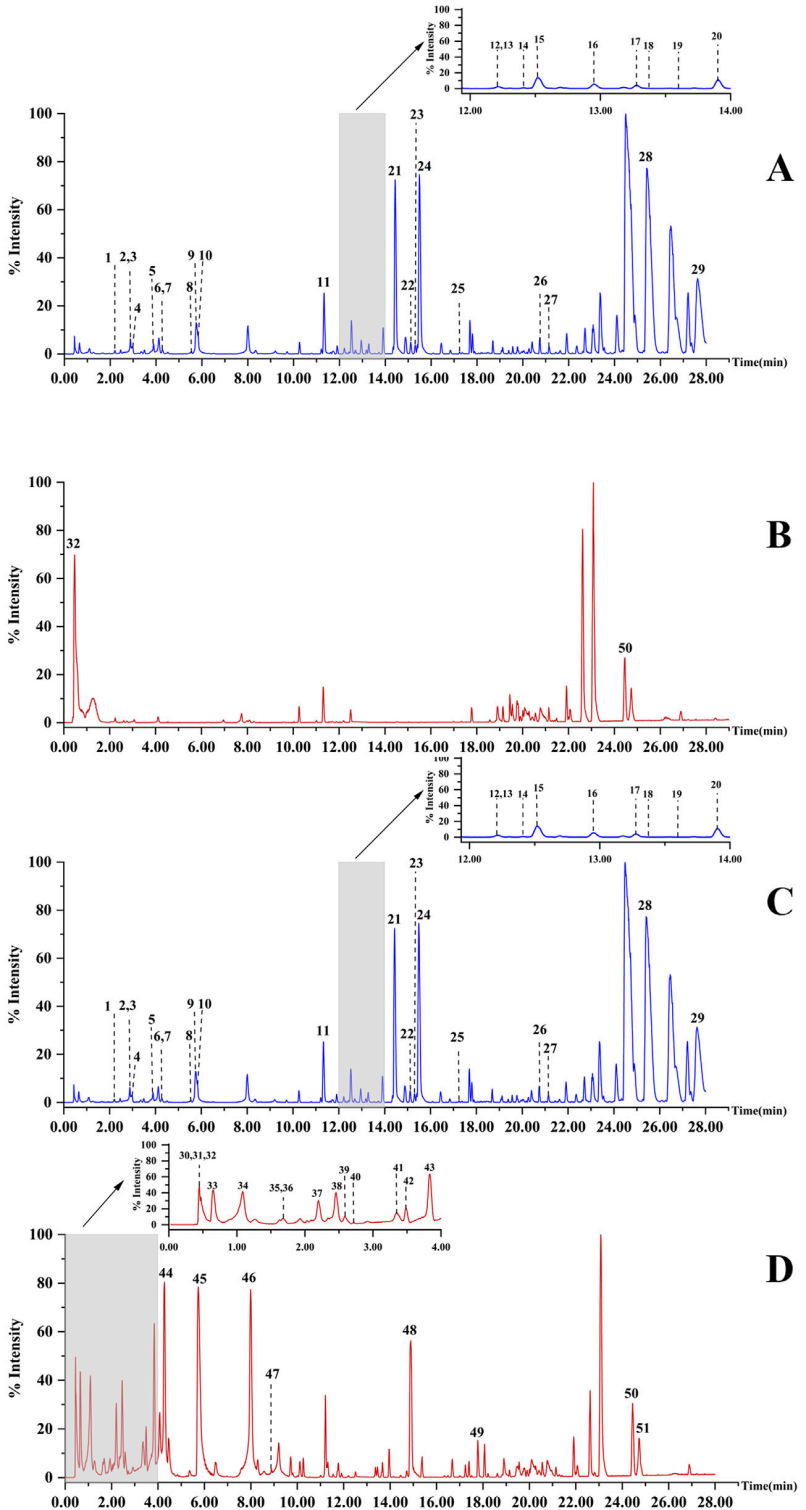
The base peak chromatograms of raw TBC in positive ionization mode **(A)** and in negative ionization mode **(B)**; the base peak chromatograms of TBC processed with Hezi-decoction for 72 h in positive ionization mode **(C)** and in negative ionization mode **(D)**.

As the soaking time increased, the Hezi decoction gradually soaked into the dried TBC (Figure 1). As a result, 51 compounds, including aconitine, 3-deoxyaconitine, benzoylaconine, aconine, chebulic acid, corilagin, and ellagic acid, were identified in raw TBC and processed samples at various time points, with 31 compounds recognized from the raw TBC. The base peak chromatograms of the unprocessed and processed TBC samples are presented (Figure 2). The identification results by the corresponding reference substances, *Aconitum* and *Terminalia* compound databases, and published literature are shown (Table 1).

**TABLE 1:** Identification of the major chemical constituents in raw and processed TBC by UPLC-Q-TOF-MS.

PeakNo.	*t*_R_ (min)	Formula	Observed *m/z*	Mass error (ppm)	Adducts	Fragment **(+/−)**	Identification	Raw TBC	Hezi-decoction-processed TBC	References
1	2.17	C_23_H_37_NO_6_	424.2701	1.7	[M + H]^+^	406.2629, 388.2514, 378.2655^,^ 356.2379	Senbusine A	✓	✓	Xu et al. (2014)
2^b^	2.87	C_22_H_31_NO_3_	358.2381	1.1	[M + H]^+^	340.2264, 330.2431	Songorine	✓	✓	Zhi M. et al. (2020), Yan et al. (2010)
3^a^ ^**,**^ ^b^	2.87	C_25_H_41_NO_9_	500.2859	1.0	[M + H]^+^	482.2770, 468.2581, 450.2487, 432.2193	Aconine	✓	✓	Zhi M. et al. (2020)
4^a^	2.97	C_22_H_33_NO_3_	360.2533	0.0	[M + H]^+^	342.2409	12-*Epi*-napelline	✓	✓	Liu (2021)
5^a^	3.89	C_24_H_39_NO_6_	438.2854	0.8	[M + H]^+^	420.2751, 356.2233, 338.2114	Neoline	✓	✓	Zhi M. et al. (2020), Yan et al. (2010)
6	4.27	C_25_H_41_NO_8_	484.2915	2.0	[M + H]^+^	452.2664, 434.2549, 420.2505, 402.2340	Pseudoaconine	✓	✓	Lei et al. (2020),
7^b^	4.32	C_26_H_41_NO_8_	496.2907	0.4	[M + H]^+^	464.2672, 428.2419, 404.2406	8-Acetyl-15-hydroxy-neoline	✓	✓	Lei et al. (2020)
8	5.55	C_26_H_41_NO_8_	496.2907	0.4	[M + H]^+^	436.2737, 418.2618, 404.2406, 376.2488, 326.2136	14-O-Acetyldelectinine	✓	✓	Lei et al. (2020)
9^b^	5.72	C_24_H_35_NO_4_	402.2640	0.3	[M + H]^+^	484.2553, 324.2276	Lucidusculine	✓	✓	He et al. (2022)
10	5.82	C_26_H_41_NO_7_	480.2960	0.8	[M + H]^+^	462.2831, 448.2675, 416.2408, 398.2360	14-Acetylneolin	✓	✓	Lei et al. (2020), Yang M. H. et al. (2014)
11^a^ ^**,**^ ^b^	11.32	C_32_H_45_NO_10_	604.3119	0.5	[M + H]^+^	572.2843, 586.2933, 554.2732, 448.2694	Benzoylaconine	✓	✓	Lei et al. (2020)
12^b^	12.21	C_32_H_43_NO_9_	586.3010	−0.1	[M + H]^+^	586.3018, 554.2754, 522.2496,	14-Benzoyl-16-ketoneoline	✓	✓	Lei et al. (2020)
13	12.21	C_34_H_47_NO_11_	646.3224	0.3	[M + H]^+^	586.2989, 554.2732, 536.2649, 522.2380	Karaconitine	✓	✓	Lei et al. (2020)
14	12.41	C_36_H_51_NO_12_	690.3490	−0.3	[M + H]^+^	640.3167, 586.2989, 554.2815	Pseudaconitine	✓	✓	He et al. (2022), Atta-Ur-Rahman et al. (2000)
15^b^	12.52	C_32_H_45_NO_9_	588.3166	−0.2	[M + H]^+^	556.3196, 536.2649, 522.2486, 504.2485, 472.2231	Benzoyldeoxyaconine	✓	✓	Zhi M. et al. (2020)
16^b^	12.98	C_32_H_43_NO_11_	618.2914	0.00	[M + H]^+^	558.2729, 554.2732, 526.2498, 508.2225	*N*-demethylmesaconine	✓	✓	Lei et al. (2020), Zhi M. et al. (2020)
17	13.27	C_32_H_43_NO_9_	586.2989	3.9	[M + H]^+^	554.2732, 536.2649, 522.2380, 504.2380	16-*Epi*-pyroaconitine	✓	✓	Li C. Y.et al. (2023)
18	13.34	C_34_H_47_NO_12_	662.3176	0.7	[M + H]^+^	630.2958, 602.2949, 572.2887	Aconifine	✓	✓	Zhi M. et al. (2020), Yang M. H. et al. (2014)
19	13.60	C_32_H_43_NO_8_	570.3080	0.6	[M + H]^+^	538.2956, 510.2789, 506.2573	Dehydrated benzoyldeoxyaconine	✓	✓	Lei et al. (2020)
20^b^	13.90	C_32_H_43_NO_10_	602.2965	0.7	[M + H]^+^	542.2783, 510.2471,478.2209	*N*-deethyldeoxyaconitine	✓	✓	Li C. Y.et al. (2023)
21^a^ ^**,**^ ^b^	14.41	C_34_H_47_NO_11_	646.3224	0.3	[M + H]^+^	596.2916, 586.2989, 554.2732, 536.2649, 506.2537	Aconitine	✓	✓	Zhi M. et al. (2020), Yang M. H. et al. (2014)
22^a^ ^**,**^ ^b^	15.11	C_36_H_49_NO_12_	688.3331	0.6	[M + H]^+^	628.3099, 596.2830, 582.3061, 550.2799,510.2868	3-Acetylaconitine	✓	✓	Lei et al. (2020)
23	15.30	C_34_H_47_NO_9_	614.3326	0.4	[M + H]^+^	582.3101, 554.3146, 522.2863	Chasmaconitine	✓	✓	Lei et al. (2020)
24^a^	15.47	C_34_H_47_NO_10_	630.3276	0.4	[M + H]^+^	598.3037, 570.3080, 538.2792,	3-Deoxyaconitine	✓	✓	Zhi M. et al. (2020), Yang M. H. et al. (2014)
25	17.24	C_36_H_51_NO_11_	674.3532	−0.4	[M + H]^+^	642.3126, 586.3159, 582.3061, 554.3112	Szechenyine	✓	✓	He et al. (2022), Sun et al. (1989)
26^b^	20.74	C_50_H_75_NO_12_	882.5383	3.8	[M + H]^+^	864.5279	10-OH-14-benzoylaconinelinoleate	✓	✓	Li C. Y.et al. (2023)
27	21.13	C_50_H_75_NO_11_	866.5422	1.0	[M + H]^+^	834.5132, 684.5283, 586.3020	14-Benzoylaconine-8-linoleate	✓	✓	Liu et al. (2023), Lei et al. (2020)
28^b^	25.43	C_50_H_75_NO_10_	850.5473	1.1	[M + H]^+^	570.3080, 538.2792, 506.2494, 494.2360	3-Deoxyaconine-8-linoleate	✓	✓	Liu et al. (2023)
29^b^	27.64	C_48_H_75_NO_10_	826.5470	0.8	[M + H]^+^	794.5314, 570.3080, 538.2792	8-*Pal*-benzoyldeoxyaconine	✓	✓	Lu et al. (2010)
30	0.45	C_7_H_12_O_6_	191.0565	1.9	[M−H]^−^	173.0437, 111.0096	Quinic acid		✓	Zhao (2021)
31	0.45	C_13_H_16_O_10_	331.0677	1.9	[M−H]^−^	271.0437, 173.0456, 169.0120, 125.0229, 111.0096	Glucogallin		✓	Zhi M. et al. (2020)
32	0.45	C_12_H_22_O_11_	341.1092	0.7	[M−H]^−^	211.0257, 179.0577, 169.0143, 161.04	Sucrose	✓	✓	Liu et al. (2023)
33^a^ ^**,**^ ^b^	0.66	C_14_H_12_O_11_	355.0315	2.2	[M−H]^−^	337.0159, 311.0416, 293.0315, 249.0407, 179.0561, 161.0456	Chebulic acid		✓	Zhou et al. (2020)
34^a^ ^**,**^ ^b^	1.08	C_7_H_6_O_5_	169.0145	1.5	[M−H]^−^	125.0229, 124.0175	Gallic acid		✓	Zhou et al., 2020; Zhao (2021)
35^b^	1.68	C_14_H_14_O_9_	325.0570	−0.4	[M−H]^−^	169.0120, 125.0244, 107.0139	Shikimic acid 5-*O*-gallate		✓	Sun Y. J. et al. (2021)
36	1.69	C_34_H_24_O_22_	783.0718	4.1	[M−H]^−^	631.0577, 450.9943	Terflavin B		✓	Zhou et al. (2020)
37	2.24	C_9_H_8_O_3_	163.0408	4.4	[M−H]^−^	119.0518	*p*-Coumaric acid		✓	Zheng et al. (2024)
38	2.45	C_20_H_20_O_14_	483.0789	1.8	[M−H]^−^	331.0663, 313.0558, 211.0268, 169.0120,	Gallic acid 3-*O*-β-D-(6′-*O*-galloyl)-glucopyranoside		✓	Nonaka and Nishioka (1983)
39	2.61	C_48_H_28_O_30_	1,083.0640	4.0	[M−H]^−^	721.0802, 600.9728, 300.9948, 273.0056	Punicalagin		✓	Pellati et al. (2013)
40	2.71	C_48_H_30_O_30_	1,085.0780	3.0	[M−H]^−^	783.0760, 450.9991	Terflavin A		✓	Zhou et al. (2020)
41	3.43	C_41_H_32_O_28_	971.1048	4.2	[M−H]^−^	633.0699, 337.0224	Neochebulagic acid		✓	Zhou et al. (2020)
42^b^	3.48	C_27_H_24_O_19_	651.0852	2.1	[M−H]^−^	481.0576, 337.0224, 275.0212, 169.0120	Chebulanin		✓	Dong et al. (2022)
43^b^	3.84	C_27_H_22_O_18_	633.0736	0.4	[M−H]^−^	300.9948, 275.0212, 169.0120	Corilagin		✓	Chen et al. (2019)
44^b^	4.26	C_27_H_24_O_18_	635.0896	0.9	[M−H]^−^	465.0681, 313.0558, 169.0120, 125.0229	1,3,6-*Tri*-*O*-galloyl-β-D-glucose		✓	Liu H. S. et al. (2021)
45^a^ ^**,**^ ^b^	5.72	C_14_H_6_O_8_	300.9992	0.8	[M−H]^−^	283.9935, 257.0120, 245.0085	Ellagic acid		✓	Zhou et al. (2020)
46^a^	7.99	C_41_H_32_O_27_	955.1085	2.8	[M−H]^−^	803.0955, 337.0159, 319.0055, 275.0212, 169.0120	Chebulinic acid		✓	Liu et al. (2023)
47	8.89	C_41_H_32_O_26_	939.1123	1.5	[M−H]^−^	769.0956, 617.0831, 169.0120, 125.0229	1,2,3,4,6-*O*-Pentagalloylglucose		✓	Zhou et al. (2020), Chen et al. (2019)
48^b^	14.89	C_30_H_48_O_6_	503.3382	0.8	[M−H]^−^	485.3278, 409.3123	Arjungenin		✓	Zhou et al. (2020)
49	17.78	C_30_H_48_O_5_	487.3443	2.8	[M−H]^−^	487.3456, 441.1952,425.3438	Arjunolic acid		✓	Chen et al. (2017), Chen et al. (2019)
50	24.45	C_16_H_32_O_2_	255.2334	1.9	[M−H]^−^	237.2270, 210.1892, 162.8387	Palmitic acid	✓	✓	Xiao et al. (2022)
51	24.72	C_18_H_34_O_2_	281.2489	0.9	[M−H]^−^	263.8383, 236.8531	Oleic acid		✓	Sun Y. J. et al. (2021), Xiao et al. (2022)

^a^ The compounds confirmed by comparison with the reference standards.

^b^ Metabolic markers between raw TBC, and Hezi-decoction-processed TBC, for 72 h.

✓The compounds identified in raw TBC, and Hezi-decoction-processed TBC, for different time points.

### 3.2 PCA and PLS-DA of raw and Hezi-decoction-processed TBC with different hours

**FIGURE 3:**
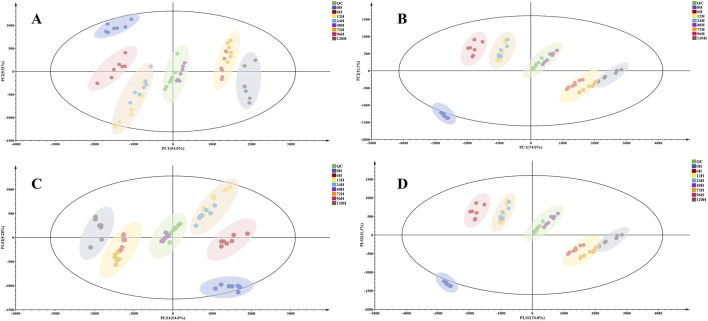
The PCA score plot of raw and processed TBC for different time points in positive **(A)** and negative **(B)** ionization mode. The PLS-DA score plot of raw and processed TBC for different time points in positive **(C)** and negative **(D)** ionization mode.

PCA and PLS-DA were used for distinguishing the variations of TBC with varying processing times, visually showing grouping trends, and revealing the metabolic differences among them (Sun et al., 2020). The PCA score plots (Figures 3A, C) could divide all the metabolites into six major groups, suggesting that the chemical profile changed as a result of processing. The raw TBC was highly different from the Hezi-decoction-processed TBC. The TBC samples with different processing time points were divided into groups and gradually moved away from the raw TBC. The results showed obvious chemical changes between raw TBC and those processed for more than 6 h. In addition, the metabolites of the TBC processed for 72 and 96 h were grouped together, indicating the chemical metabolites were more similar.

The outcomes of PLS-DA discrimination aligned well with the PCA model, resulting in the categorization of all data into six distinct categories (Figures 3B, D). And the samples processed for a longer time were located closer, indicating a good accordance with the PCA model. The results of PCA and PLS-DA preliminarily revealed that the metabolites of TBC changed clearly from 0 to 72 h, but the variations of TBC metabolites gradually stabilized after 72 h.

### 3.3 Discovery of Hezi-decoction-processed-associated metabolic markers in raw and processed TBC with different hours

OPLS-DA was carried out to identify variables that result in group separation between raw and processed TBC. The primary focus was on raw TBC and TBC that had undergone processing for 72 h, as these groups were selected to elucidate the metabolic markers. OPLS-DA was used for comparison of metabolic changes.

**FIGURE 4:**
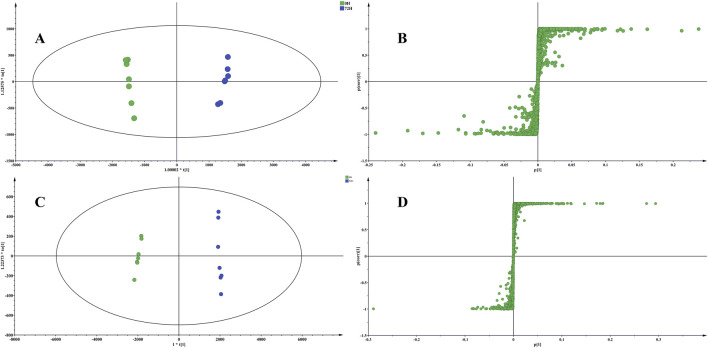
The OPLS-DA score plot based on raw TBC in positive **(A)** and negative ionization mode **(C)**; The S-polt based on TBC processed with Hezi-decoction for 72 h in positive **(B)** and negative ionization mode **(D)**.

**FIGURE 5:**
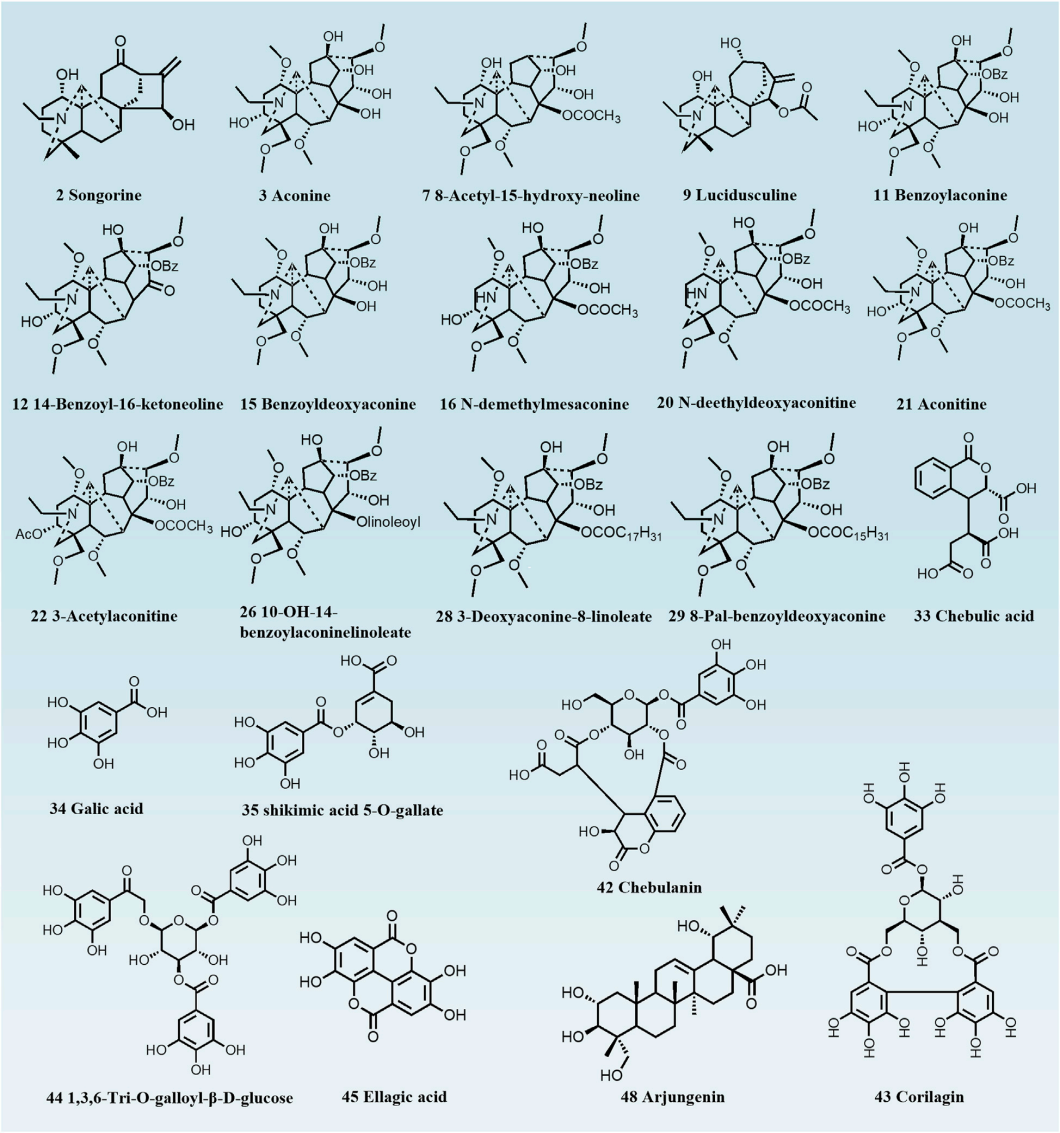
Chemical structures of metabolic markers of unprocessed and Hezi-decoction-processed TBC for 72 h.

Distinct group separation was evident in both OPLS-DA score plots (Figures 4A, C), highlighting a vast disparity in the chemical profiles of raw TBC and TBC processed for 72 h. The associated S-plot (Figures 4B, D) illustrated that the ions positioned away from the origin played a crucial role in differentiating between raw TBC and the processed TBC. Based on the results of the S-plots and the VIP value (VIP greater than 1) obtained from OPLS-DA analysis, and the corresponding metabolites were detected. A total of 22 metabolites, including aconine, aconitine, benzoylaconine, chebulic acid, gallic acid, and corilagin, can proficiently distinguish between raw and processed TBC with different processing times (Table 1; Figure 5).

### 3.4 Determination of main alkaloids in raw and Hezi-decoction-processed TBC

#### 3.4.1 Method validation

The analytical methodology employed in this investigation was validated according to the guidelines set forth by the Chinese Pharmacopoeia 2020. The parameters of linearity, accuracy, precision, and stability were assessed through the analysis of samples and various concentrations of standards. Variability was quantified by conducting analyses on six replicates. The relative standard deviation (RSD) was computed to assess precision, stability and repeatability. Additionally, six samples of raw TBC were prepared and analyzed on the same day to assess repeatability. Stability was examined at ambient temperature, with analyses conducted at intervals of 0, 4, 8, 12, 18, and 24 h. The recovery was determined by calculating the recovery percentages of benzoylaconine, aconitine, and 3-deoxyaconitine in the spiked samples. Six parallel samples were prepared for each concentration.

The three compounds demonstrated excellent linearity (r^2^ = 1.00) across the specified test ranges. The RSDs for intra-day, inter-day, stability, and repeatability assessments were observed to be within the ranges of 0.32%–0.45%, 0.71%–0.76%, 0.98%–1.22%, and 2.41%–2.89%, respectively. The recovery rates for the five tannin acids were found to range from 97.28% to 101.48% (Tables 2, 3). Consequently, the accuracy and feasibility of the method were demonstrated by all these results.

**TABLE 2:** Regression equations, linear range, precision, repeatability, and stability of the method of three alkaloids.

Compound	t_R_ (min)	Regression equation	*r* ^2^	Linear range (mg/mL)	Precision		Repeatability	Stability
					Intraday (n = 6)	Interday (n = 6)		
Benzoylaconine	9.03	*y* = 5,094, 728.96 *x* + 1,911.80	1.0000	0.0160–0.1599	0.45	0.75	2.81	0.98
Aconitine	19.26	*y* = 6,210, 378.77 *x* − 30,514.45	1.0000	0.0298–0.4775	0.32	0.76	2.41	1.22
3-deoxyaconitine	32.92	*y* = 6,764, 678.43 *x* − 28,855.82	1.0000	0.0519–0.2076	0.35	0.71	2.89	1.11

**TABLE 3:** Recovery of the method of three alkaloids.

Compound	Amount (mg) (n = 6)	Added amount (mg) (n = 6)	Detected amount (mg) (n = 6)	Recovery (%)(n = 6)	RSD (%) (n = 6)
Benzoylaconine	0.3379	0.3359	0.6764	101.48	0.44
	0.3379	0.3359	0.6776		
	0.3381	0.3359	0.6807		
	0.3380	0.3359	0.6798		
	0.3379	0.3359	0.6796		
	0.3380	0.3359	0.6788		
Aconitine	0.6121	0.6160	1.2071	97.28	0.85
	0.6123	0.6160	1.2154		
	0.6119	0.6160	1.2037		
	0.6121	0.6160	1.2144		
	0.6122	0.6160	1.2108		
	0.6118	0.6160	1.2165		
3-deoxyaconitine	0.4728	0.4760	0.9410	100.77	1.21
	0.4727	0.4760	0.9547		
	0.4726	0.4760	0.9556		
	0.4726	0.4760	0.9551		
	0.4726	0.4760	0.9523		
	0.4726	0.4760	0.9542		

#### 3.4.2 Content of main alkaloids in raw and processed TBC

The accurate concentrations of three alkaloids in the samples of processed TBC at different time points were determined using the regression equations (Table 4). The result indicated that the MDAs and DDAs showed a gradual decrease and tended to be flat after 48 h of processing in general. The MDAs levels were lower than 0.0162% after 48 h of processing, and then they fluctuated between 0.0149% and 0.0162%. The MDAs levels was 0.0149% in 72 h. The DDAs levels were lower than 0.0900% after 24 h, and then it fluctuated between 0.0851% and 0.0879%. Nevertheless, the DDAs levels had a minor reduction to 0.0852% after 72 h, followed by a further increase. The MDA and DDAs levels were 0.0149% and 0.0852% in 72 h, respectively. Combined with the outcomes of UPLC and multivariate statistical analysis, a processing time of 72 h was further found to be the appropriate time for toxicity attenuation and efficacy reservation of TBC.

**TABLE 4:** The contents of three alkaloids and five tannic acids (n = 3).

Time (h)		0 h	6 h	12 h	24 h	48 h	72 h	96 h	120 h
Content of five tannic acids (mg/g)	Gallic acid	0	1.3365	1.6216	2.0791	5.7362	8.9706	9.6056	9.5152
	Corilagin	0	1.3922	1.6892	2.1658	5.9752	9.3444	10.0058	9.9117
	1,2,3,4,6-*O*-Pentagalloylglucose	0	0.9233	1.1797	0.9734	1.3420	1.2438	1.2303	1.1672
	Chebulinic acid	0	1.2537	2.0556	2.0175	4.5133	5.7582	5.6796	5.1351
	Ellagic acid	0	0.4123	0.6420	0.6944	1.3839	3.1160	3.3610	3.1854
Content of alkaloids (mg/g)	Benzoylaconine	0.3380	0.3049	0.2153	0.1689	0.1542	0.1485	0.1617	0.1605
	Aconitine	0.6120	0.5618	0.5364	0.5846	0.5192	0.5403	0.5407	0.5402
	3-deoxyaconitine	0.4726	0.4316	0.3501	0.3155	0.3595	0.3117	0.3104	0.3119
Proportion of MDAs (%)		0.0338	0.0305	0.0215	0.0169	0.0154	0.0149	0.0162	0.0160
Proportion of DDAs (%)		0.1085	0.0993	0.0887	0.0900	0.0879	0.0852	0.0851	0.0852

### 3.5 Determination of main tannin acids in raw and Hezi-decoction-processed TBC

#### 3.5.1 Method validation

Linearity, accuracy, precision, and stability were validated as mentioned above. The mixed solutions for five tannin acids were utilized in the regression analyses correlating peak areas with the concentrations. The five tannin acids exhibited excellent linearity across the tested ranges. R^2^ were 0.9991, 0.9993, 0.9997, 0.9991 and 0.9998, respectively. The RSDs for intra-day, inter-day, stability, and repeatability were in the ranges of 0.47%–1.94%, 1.69%–2.83%, 0.14%–3.16%, and 1.07%–2.53%, respectively. Recoveries of five tannin acids varied between 99.33% and 101.20% (Tables 5, 6). The accuracy and feasibility of the method were demonstrated by all these results.

**TABLE 5:** Regression equations, linear range, precision, repeatability, and stability of the method of five tannic acids.

Compound	*t*_*R*_ (min)	Regression equation	*r* ^2^	Linear range (mg/mL)	Precision		Repeatability	Stability
					Intraday (n = 6)	Interday (n = 6)		
Gallic acid	9.62	*y* = 24,057, 247.70 *x−*696,722.58	0.9991	0.0508–1.6240	0.71	1.73	2.53	3.16
Corilagin	34.66	*y* = 22,037, 065.06 *x* + 380,727.47	0.9993	0.0506–0.8096	0.73	1.89	2.53	3.16
1,2,3,4,6-*O*-Pentagalloylglucose	56.46	*y* = 6,379, 898.56 *x−*87,856.82	0.9997	0.0296–0.2960	1.94	2.83	2.29	0.28
Chebulinic acid	63.15	*y* = 9,886, 8888.02 *x−*120, 244.83	0.9991	0.0510–0.8160	0.47	1.69	1.07	0.14
Ellagic acid	66.969	*y* = 120,637, 245.16 *x−*156, 841.90	0.9998	0.0254–0.4563	0.84	1.60	2.24	1.19

**TABLE 6:** Recovery of the method of five tannic acids.

Compound	Amount (mg) (n = 6) (mg)	Added amount (mg) (n = 6)	Detected amount (mg) (n = 6)	Recovery (%) (n = 6)	RSD (%) (n = 6)
Gallic acid	10.2520	10.2610	20.4051	101.20	3.70
	10.2561	10.2610	20.4193		
	10.2530	10.2610	20.4581		
	10.2540	10.2610	20.5101		
	10.2581	10.2610	21.0174		
	10.2571	10.2610	21.0229		
Corilagin	3.7256	3.7320	7.4350	99.33	2.46
	3.7276	3.7320	7.4179		
	3.7295	3.7320	7.5680		
	3.7273	3.7320	7.3571		
	3.7287	3.7320	7.5091		
	3.7284	3.7320	7.3225		
1,2,3,4,6-*O*-Pentagalloylglucose	1.6522	1.6550	3.3418	100.47	3.32
	1.6520	1.6550	3.3832		
	1.6517	1.6550	3.3371		
	1.6513	1.6550	3.2363		
	1.65252	1.6550	3.2602		
	1.6523	1.6550	3.3298		
Chebulinic acid	9.7160	9.7130	19.0769	99.82	1.94
	9.7150	9.7130	19.3910		
	9.7121	9.7130	19.5752		
	9.7140	9.7130	19.5801		
	9.7179	9.7130	19.3643		
	9.7150	9.7130	19.4757		
Ellagic acid	3.8878	3.8820	7.7420	100.72	2.56
	3.8882	3.8820	7.7310		
	3.8854	3.8820	7.7110		
	3.8862	3.8820	7.7560		
	3.8866	3.8820	7.8840		
	3.8870	3.8820	7.9580		

#### 3.5.2 Content of five tannin acids in raw and processed TBC

The accurate concentrations of five tannin acids in the samples of processed TBC at different time points were determined using the regression equations obtained from their curves. The findings demonstrated that the content of five tannin acids gradually escalated from 6 to 12 h, followed by a substantial increase from 12 to 72 h. Finally, the contents increased slowly to a steady level after 72 h. The contents of gallic acid, corilagin, 1,2,3,4,6-*O*-pentagalloylglucose, chebulinic acid, and ellagic acid were 8.9706, 9.3444, 1.2438, 5.7582, and 3.1160 mg/g, respectively (Table 4). Based on the multivariate statistical analysis and content determination of three alkaloids and five tannin acids, 72 h is demonstrated to be the appropriate time for toxicity attenuation and efficacy reservation of TBC.

### 3.6 *In situ* metabolite profiling of TBC and its processed products by DESI-MSI

*In-situ* molecular detection was performed in simulated industrial segments of TBC utilizing DESI-MS. This approach aimed to examine the alterations in eight potential metabolic markers at various processing times of TBC.

**FIGURE 6:**
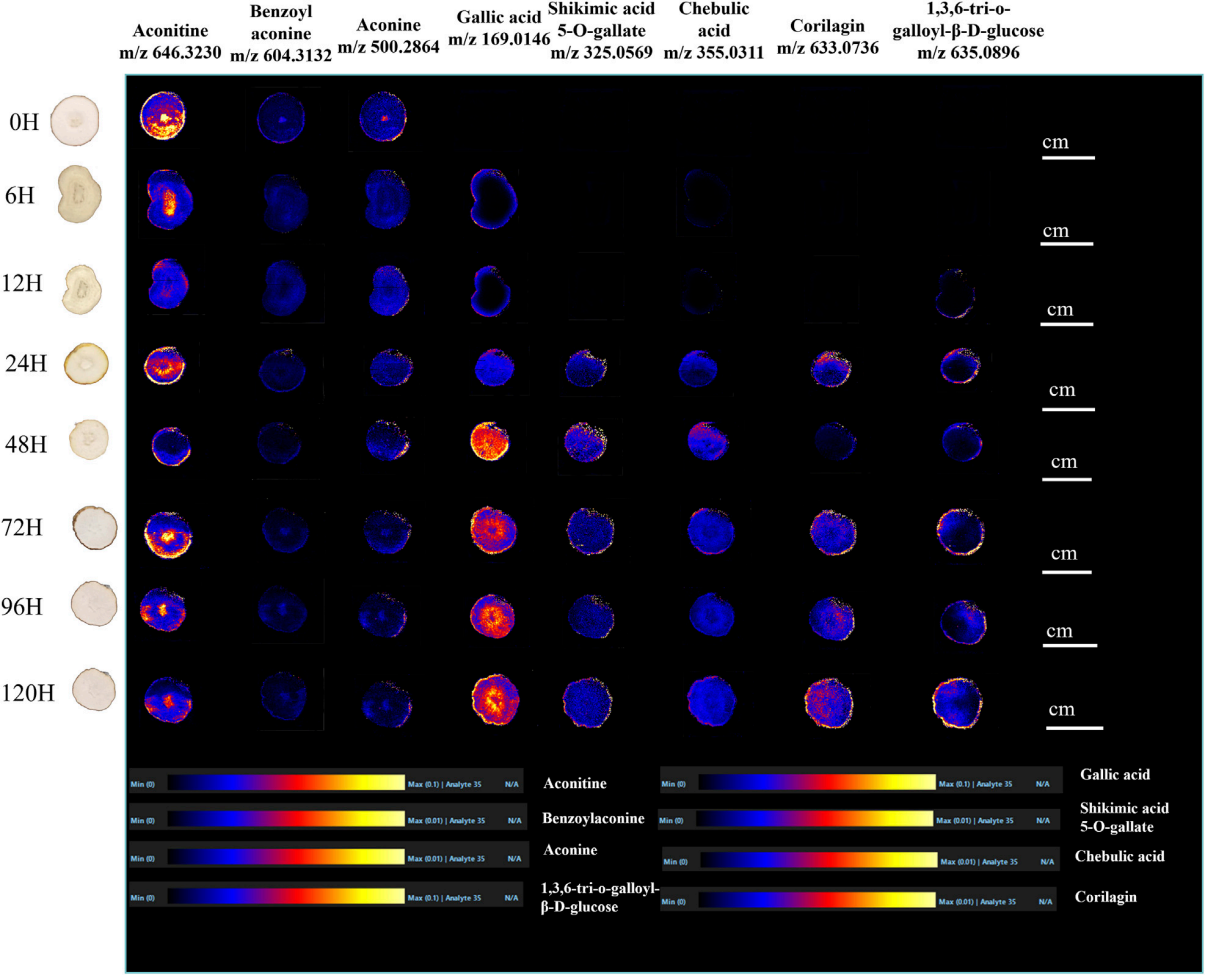
DESI-MS images of eight major metabolic markers in raw and processed TBC for 72 h.

The MS images (Figure 6) illustrate the presence of three alkaloids: aconitine (*m/z* 646.3230), benzoylaconine (*m/z* 604.3132), and aconine (*m/z* 500.2864), with assessed in positive mode (Sun C. L. et al., 2021). The results of DESI-MSI visualization indicated that with prolonged processing time, the response values of the three alkaloids gradually decreased and then tended to be stable. A slight decrease was observed in the response values of benzoylaconine (MDA) and aconine (NDA) from 0 to 12 h. The response values significantly decreased from 12 to 72 h. Finally, the response values stabilized after 72 h. Conversely, the response values of aconitine (DDA) declined slightly from 0 to 6 h and from 6 to 12 h. Then, they fluctuated from 12 to 72 h, with increments observed from 12 to 24 h and from 48 to 72 h, whereas a decrease occurred from 24 to 48 h. After 72 h, the response value decreased slightly and became stable.

In addition, five tannin acids, including gallic acid (m/z 169.0146), shikimic acid 5-*O*-gallate (m/z 325.0569), chebulic acid (m/z 355.0311), corilagin (m/z 633.0736), and 1,3,6-tri-*O*-galloyl-β-D-glucose (m/z 635.0896), were presented in negative mode (Mao et al., 2021). The results of DESI-MSI visualization revealed that with prolonged processing time, the response values of tannin acids gradually increased and then stabilized. In particular, the response values slowly increased from 6 to 12 h, exhibited a considerable increase after 24 h, and then steadied after 72 h. Additionally, the response values of shikimic acid 5-*O*-gallate, corilagin, and 1,3,6-tri-*O*-galloyl-β-D-glucose were too low to detect at 6 and 12 h. The results of DESI-MSI were consistent with those of content determination experiments, indicating its reliability.

## 4 Discussion

TBC has been employed in Tibetan medicine for millennia, owing to its remarkable pharmacological efficacy and toxicity, particularly for treating fever, arthritis, and traumatic injuries. Similar to Caowu (*Aconitum kusnezoffii* Reichb.), which also belongs to the genus *Aconitum*, it demonstrates a correlation between its therapeutic efficacy and toxicity, linked to the presence of high-toxicity DDAs such as aconitine and 3-deoxyaconitine, alongside moderate-toxicity MDAs such as benzoylaconine (Liu et al., 2017; Tang et al., 2021). This is why TBC is used with a processing method that attenuates toxicity and preserves efficacy to ensure clinical safety. Known as the “king of medicines” for its diverse pharmaceutical and antidotal effects, Hezi is widely employed in Tibetan medical formulations as a particularly good remedy for aconite poisoning. According to ancient literature and modern studies, the Hezi-decoction-processed method is one of the distinctive traditional processing methods of Tibetan medicine. (Bao and Na, 2020; Zhang et al., 2021). Nevertheless, the overall variability of metabolites in Hezi-decoction-processed TBC is still unclear. UPLC-Q-TOF-MS-based metabolomics was utilized for uncovering biomarkers that could distinguish between raw and processed TBC. Combining quantitative methods and DESI-MSI visualization demonstrated that the dynamic changes of biomarkers in TBC were supervised during processing time.

UPLC-Q-TOF-MS was carried out to identify toxic and effective compounds between unprocessed TBC and processed samples with Hezi decoction for different times. Fifty-one compounds, including aconitine, 3-deoxyaconitine, benzoylaconine, aconine, chebulic acid, corilagin, and ellagic acid, were identified from unprocessed and processed samples, of which 31 were discernible from the unprocessed TBC.

In combining metabolomics based on UPLC-Q-TOF-MS and multivariate statistical analysis, a total of 22 metabolites, including aconine, aconitine, benzoylaconine, chebulic acid, gallic acid, and corilagin, can proficiently distinguish between raw and processed TBC. UPLC was first performed to quantify variation in the contents of one MDA (benzoylaconine) and two DDAs (aconitine and 3-deoxyaconine). Eight processing times were defined and the UPLC data showed that two DDAs (aconitine and 3-deoxyaconine) decreased and then remained about 0.0852% from 0 to 72 h. With prolonged processing time, MDA (benzoylaconine) was reduced slowly from 0 to 12 h, and it showed clearly decreases from 12 to 24 h. Then, it fluctuated between 0.0149% and 0.0162% after 24 h. The MDA and DDAs levels were 0.0149% and 0.0852% in 72 h, respectively. In addition, the contents of five tannin acids (gallic acid, corilagin, 1,2,3,4,6-*O*-pentagalloylglucose, chebulic acid, and gallic acid) showed a slowly increasing trend from 6 to 12 h. From 12 to 72 h, the content increased clearly. With the expendied processing time, the raw TBC was completely soaked by Hezi decoction, and the contents increased significantly. Finally, the contents became stable after 72 h. The contents of gallic acid, corilagin, 1,2,3,4,6-O-pentagalloylglucose, chebulinic acid, and ellagic acid were 8.9706, 9.3444, 1.2438, 5.7582, and 3.1160 mg/g, respectively.

**FIGURE 7:**
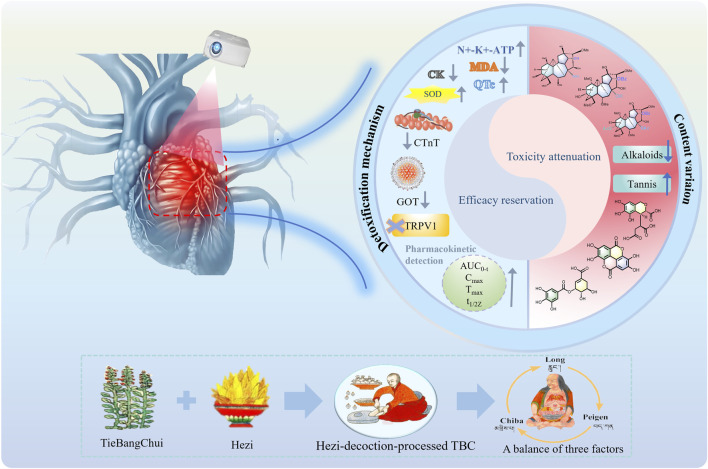
The mechanism of efficacy reservation and toxicity attenuation in the Hezi-decoction-processed TBC.

Toxicity and efficacy are mutually dependent in TBC because of its high levels of highly toxic DDAs and moderately toxic MDAs. Hezi is rich in high levels of tannin acids such as gallic acid, ellagic acid, chebulinic acid, corilagin and punicalagin, which may improve anti-arthritic effects and reduce cardiotoxicity caused by TBC (Gao et al., 2024). After being processed with Hezi decoction, the levels of DDAs and MDAs, the toxicity and efficacy components in TBC, were properly reduced. And the addition of tannin acids can effectively reduce the contents of major alkaloids to a certain extent. Meanwhile, tannin acids can inhibit dissolution and hydrolysis, so alkaloids can be slowly hydrolyzed and have a slow release effect *in vivo* (Liu et al., 2013; Liu et al., 2015). Pharmacokinetic studies proved that the active ingredients of *Chebulae Fructus* formed complexes with alkaloids in the blood and blood-rich tissues, slowing down distribution, elimination and detoxification (Wang et al., 2002). The Hezi-processed method has the potential to decrease both the concentration and the rate of absorption and elimination of various alkaloids in Caowu, thereby extending their duration of action. The mechanism of detoxification and effect preservation of Hezi-decoction-processed TBC was revealed from the viewpoint of material basis (Zhang et al., 2021; Zhi M. R. et al., 2020). In addition, it is related to compatibility effects. Due to its large amounts of tannin acids, Hezi has cardioprotective, anti-inflammatory and antioxidant (Upadhyay et al., 2014). Toxicological studies showed that the *T. chebula* extract can mitigate injury of aconitinc on myocardium, and it has a protecting effect on damaged myocardium (Zhang, 2010). Gallic acid and ellagic acid, characteristic components of *T. chebula*, synergistically reduce cardiotoxicity caused by mesaconitine and benzoylmesaconitine (Han et al., 2022). Academics found that Caowu administered together with Hezi could significantly reduce serum CK, GOT and reduce myocardial tissue Ca^2+^ content to increase Na^+^ -K^+^ -ATPase activity compared to unprocessed Caowu. Besides, it is reported that Caowu processed with Hezi decoction can block TRPV channel and prevent cardiotoxicity (Han et al., 2022; Song et al., 2024). In addition, corilagin contained in*Terminalia chebula* has significant anti-inflammatory effects. Its intrinsic anti-inflammatory mechanism involves a significant reduction in the production of pro-inflammatory cytokines and mediates TNF-α, IL-1β, IL-6, NO (iNOS), and COX-2 nuclear translocation by blocking NF-κB at the protein and gene levels (Zhao et al., 2008). It is reported that gallic acid, 2,3,6-tri-O-gallate-β-D-glucose, and arjunic acid, which are found in *T. chebula*, have anti-inflammatory activity by inhibiting iNOS and COX-2 activity at the cellular level and effectively reducing nitric oxide production (Yang Y. et al., 2014). Furthermore, TBC co-administered with Hezi was able to attenuate cardiotoxicity associated with TBC while preserving its efficacy in treating rheumatoid arthritis. The compatibility of TBC and HZ has been shown to diminish the elevation in heart rate and the prolongation of the QTc interval that TBC typically induces. The synergism of TBC and HZ reduced serum cTnT and cardiac MDA levels, raised cardiac SOD levels, and effectively alleviated paw swelling to maintain the anti-rheumatoid arthritis efficacy of TBC (Liu et al., 2023; Zhang et al., 2017). Metabolomics findings demonstrated that Hezi can decrease the levels of glutamic oxalic aminotransferase, glutamic pyruvic aminotransferase, myoglobin, and troponin in the serum of rats induced by Caowu (Liu et al., 2018). In summary, effectively reducing toxicity and preserving the effect of TBC processed with Hezi decoction are likely attributable to the synergistic effects of the two aforementioned factors (Figure 7).

MSI is a molecular imaging technique that is based on mass spectrometry. It enables the visualisation of the geographical and temporal distribution of metabolites without compromising tissue integrity, providing important situ information for plant metabolomics research. A method without embedding and complex treatment of samples made the DESI-MSI sample preparation process simpler. DESI-MSI was applied between raw and Hezi-decoction-processed TBC with different processed times to achieve an integrative understanding of the distribution and accumulation of metabolic markers. The results of DESI-MSI visualisation indicated that the response values of the three alkaloids gradually decreased and then stabilised with the extension of processed time. Hezi decoction infiltrated raw TBC, resulting in the response values of benzoylaconine (MDAs) and aconine (NDAs) to slightly decrease from 0 to 12 h. Then, the response values declined clearly from 12 to 72 h. Finally, raw TBC was completely soaked by Hezi decoction, and the response values became stable after 72 h. Different from the other two alkaloids, the response values of aconitine (DDA) gradually decreased in fluctuation with prolonged processed time and became stable after 72 h after a slight decrease. The results of five tannic acids, including gallic acid, shikimic acid 5-*O*-gallate, chebulic acid, corilagin, and 1,3,6-tri-*O*-galloyl-β-D-glucose, showed that with the extension of processed time, the response values gradually increased and then tended to be stable. Combined with the DESI-MSI results of alkaloids and tannic acids, the change trend of response values over time was consistent with the process of dried TBC soaked in Hezi decoction. DESI-MSI was effectively used to visualise the distribution features of biomarkers between unprocessed and Hezi-decoction-processed TBC taken at various time points. The results of DESI-MSI were consistent with those of content determination. Consequently, the multivariate statistical analysis, content determination of three alkaloids and five tannin acids, and DESI-MSI, revealed that 72 h was found to be conducive to the attenuation of toxicity and the preservation of efficacy. This integrated strategy not only revealed the dynamic changes in metabolic markers of Hezi-decoction-processed TBC, but also preserved efficacy and attenuated toxicity, establishing an effective quality control and evaluation procedure to ensure the safety of TBC.

From the perspective of processing, this study revealed the dynamic changes and the distribution of metabolic markers in the process of efficacy preservation and toxicity reduction of Hezi-decoction-processed TBC. This result should be further verified in combination with pharmacological experiments and the dose-toxin-effect relationship *in vivo* to provide a scientific basis for further elucidating the principle of Hezi-decoction-processed TBC for detoxification, thereby further optimising the processing technology’s quality standard to ensure the safety and effectiveness of the clinical use of TBC.

## 5 Conclusion

This research developed a metabolomics approach utilising UPLC-Q-TOF-MS in conjunction with DESI-MSI visualisation and quantitative methodologies to monitor the dynamic changes in biomarkers associated with TBC throughout various processing times. A total of fifty-one compounds were discernible from unprocessed and processed samples, of which 31 were discernible from unprocessed TBC. Through the integration of UPLC-Q-TOF-MS with multivariate statistical analysis, 22 metabolic markers, such as aconine, aconitine, benzoylaconine, chebulic acid, gallic acid, and corilagin, can proficiently distinguish between raw and processed TBC with different processing times. The results of content determination of three alkaloids and five tannins showed that they were stabilized at 72 h. The MDA and DDAs levels were 0.0149% and 0.0852% in 72 h, respectively. The contents of gallic acid, corilagin, 1,2,3,4,6-O-pentagalloylglucose, chebulinic acid, and ellagic acid were 8.9706, 9.3444, 1.2438, 5.7582, and 3.1160 mg/g, respectively. The distribution and accumulation of metabolic markers during processing were investigated by DESI-MS. The results of DESI-MSI were consistent with those of content determination. Combined with the multivariate statistical analysis, content determination of three alkaloids and five tannin acids and DESI-MSI, 72 h is demonstrated to be the appropriate time for toxicity attenuation and efficacy reservation of TBC. The reliability of the Hezi-decoction processing method, as a distinctive traditional processing method of Tibetan medicine, was verified in terms of efficacy preservation and toxicity attenuation and the identification of markers in Hezi-decoction-processed TBC, establishing an effective quality control and evaluation procedure to ensure the safety of TBC. In addition, this approach facilitates a comprehensive and effective understanding of the processing mechanisms of Aconitum and other toxic traditional Chinese medicines.

## Data Availability

The original contributions presented in the study are publicly available. This data can be found here: https://www.ebi.ac.uk/metabolights/MTBLS12362

## Abbreviations

DDAs, diester-diterpenoid alkaloids; DESI-MSI, desorption electrospray ionization mass spectrometry imaging; Hezi, *Terminalia chebula* Retzs; HDI, high-definition imaging; HPLC, high-performance liquid chromatography; MDAs, monoester-diterpenoid alkaloids; NDAs, non-esterified diterpene alkaloids; OPLS-DA, orthogonal partial least-square discriminant analysis; PCA, principal component analysis; PLS-DA, partial least-square discriminant analysis; RSD, relative standard deviation; TBC, *Aconitum pendulum* Busch; UPLC, ultra-high-performance liquid chromatography; UPLC-Q-TOF-MS, ultra-high-performance liquid chromatography-quadrupole time-of-flight mass spectrometry; VIP, variable importance for projection.
